# Digital Twin Network-Based 6G Self-Evolution

**DOI:** 10.3390/s25113543

**Published:** 2025-06-04

**Authors:** Yuhong Huang, Mancong Kang, Yanhong Zhu, Na Li, Guangyi Liu, Qixing Wang

**Affiliations:** 1 China Mobile Research Institute, Beijing 100053, China; huangyuhong@chinamobile.com (Y.H.); zhuyanhongyjy@chinamobile.com (Y.Z.); linawx@chinamobile.com (N.L.); liuguangyi@chinamobile.com (G.L.); wangqixing@chinamobile.com (Q.W.); 2 School of Electronics and Information Engineering, Beijing Jiaotong University, Beijing 100091, China; 3 ZGC Institute of Ubiquitous-X Innovation and Application, Beijing 100191, China

**Keywords:** 6G, digital twin network, pre-validation environment, graph neural network

## Abstract

Digital twins (DTs) will revolutionize network autonomy. Recent studies have promoted the idea of a DT-native 6G network, deeply integrating DTs into mobile network architectures to improve the timeliness of physical–digital synchronization and network optimizations. However, DTs have mainly acted just as a tool for network autonomy, leading to a gap regarding the ultimate goal of network self-evolution. This paper analyzes future directions concerning DT-native networks. Specifically, the proposed architecture introduces a key concept called “future shots” that gives accurate network predictions under different time scales of self-evolution strategies for various network elements. To realize the future shots, we propose a long-term hierarchical convolutional graph attention model for cost-effective network predictions, a conditional hierarchical graph neural network for strategy generation, and methods for efficient small-to-large-scale interactions. The architecture is expected to facilitate high-level network autonomy for 6G networks.

## 1. Introduction

Emerging technologies and more flexible and open network architectures will be integrated into future mobile communication networks to efficiently meet the demands of rapidly evolving applications such as the low-altitude economy, immersive communications, mobile large-scale AI models, etc. [1]. Network parameters will become increasingly complex, network environments will be more heterogeneous in terms of vendors and resources, and service traffic will be denser and more diverse. These trends will significantly increase the complexity and cost of network operation optimization and maintenance, necessitating efficient network autonomy methods. Against this backdrop, Network Digital Twins (NDTs) have become particularly critical [2]. By intelligently mapping the physical network into a digital space, NDTs enable autonomous network inference and intervention [3], facilitating self-configuration, self-healing, self-optimization, and—eventually—self-evolution.

Due to the established architecture of 5G networks, current industry explorations of NDTs primarily adopt an external NDT approach to realize fragmented network autonomy [4], which is characterized by the following aspects. Firstly, external NDT is often employed to address specific issues, such as wireless coverage pre-verification [5,6], network traffic prediction [7], and user mobility forecasting [8]. For example, the authors of [5] used DTs to model the wireless channels of a reconfigurable intelligent surface (RIS)-enabled environment so as to pre-verify the RIS performance. The authors of [6] established a DT of a large-scale wireless environment to train a reinforcement learning-based base station cooperative strategy for coverage optimization. The authors of [7] proposed a prediction method for the future background traffic of a typical local area network for NDTs, which can be used for network resource management.

Secondly, existing external NDT solutions primarily rely on large-scale periodically collected physical data to train inference models offline [9]. For example, the authors of [10] used historical data and neural networks to build an NDT where the data are periodically updated, leading to a virtual interaction environment for a DRL-based network slice strategy. The authors of [11] trained a generalized federated reinforcement learning approach in a DT of an unmanned aerial vehicle-assisted mobile edge computing system to improve learning efficiency. The authors of [12] proposed a DT of a 5G core network to reduce the exploration cost of a DRL-based slice admission control strategy.

In summary, external NDT solutions typically require siloed, independent designs in different autonomy use cases, leading to high expert costs and a lack of comprehensive consideration of multiple factors. In addition, the external data collection paradigm for physical networks results in high transmission costs, long model update cycles, and the inability to optimize the physical network in real time. Consequently, this external approach can only serve network operation and maintenance use cases with low real-time requirements, failing to support network optimizations in the running state that demand high real-time performance.

To address the limitations of external NDTs, researchers have proposed the integration of NDTs into 6G network design from the outset, forming a DT-native network architecture. This DT-native paradigm embeds an NDT as an internal function within different network layers or network elements, such as an Operations, Administration, and Maintenance (OAM) platform, a Radio Access Network (RAN), and gNodeB (gNB), enabling real-time data synchronization, dynamic modeling, and closed-loop control to serve both operational and running states. For example, the authors of [13] designed a service-based DT-native network architecture. Specifically, a centralized DT is connected to a bus with other network functions, whose internal DTs as well as physical data can be efficiently collected through the bus. They also proposed an efficient model construction and an intelligent orchestration and management method for the service-based DT-native network. Similarly, ref. [14] designed a service-based architecture for a DT-native network, and they proposed a new data pipeline mechanism and a gray-box modeling method for efficient real-time data acquisition and modeling.

However, current studies on DT-native networks primarily focus on architecture design and basic enabling technologies like modeling and data collection. They fall short of achieving real full-life-cycle management, which can eventually realize a self-evolution network. This is a highly challenging and complex endeavor. Firstly, it is essential to accurately and cost-effectively predict the overall network status and the status of individual network elements over various time periods. This enables the timely detection of potential issues and large-to-small time-scale performance degradation trends. Nevertheless, the presence of numerous network elements—including network nodes, terminals, services, and environmental factors—renders short-time-scale and long-time-span predictions computationally prohibitive. Additionally, predicting each network element necessitates considering multiple other elements factors, further increasing prediction costs. Secondly, network evolution strategies range from minor parameter optimizations to major technological changes, such as the introduction of cell-free or terahertz technologies. Precisely selecting appropriate network strategies for different scenarios to evolve the network remains a key challenge. Finally, network interactions should occur across different architectural layers in non-real-time, near-real-time, and real-time modes, posing further implementation challenges.

To address these challenges, this paper proposes a self-evolution enabled DT-native network framework that is akin to architecture-centric studies such as [15,16,17,18]. To enhance reader understanding and credibility, we provide potential implementation directions for key modules, such as the cross-time hierarchical graph attention model (CTHGAM) and long-term hierarchical convolutional graph attention model (LTHCGAM), demonstrating that the proposed architecture is theoretically feasible. The main contributions of this paper are summarized as follows:

- First, an architecture is proposed for the DT-native network to realize 6G self-evolution, which includes a new concept of “future shot” for predicting future states and evolution strategy performance.
- Second, we propose a full-scale network prediction method for requirement predictions and strategy validations, which include a CTHGAM model for generating predicting strategy and an LTHCGAM for generating predicting results.
- Third, a method for generating an evolution strategy is proposed, which incorporates a conditional hierarchical Graph Neural Network (GNN) for selecting evolution elements and Large Language Model (LLM) for giving evolution strategy models. In addition, we design efficient hierarchical virtual–physical interaction strategies.
- Finally, we analyze four potential applications of the proposed DT-native network.

The remainder of our work is organized as follows. Section 2 gives the proposed architecture of the DT-native network. Section 3 gives the detail of three key technologies of the DT-native network. Section 4 gives several potential use cases of the proposed DT-native network. Finally, we conclude this paper in Section 5.

## 2. Architecture of DT-Native Network

This section gives the architecture of DT-native network, which gives a detailed understanding of what reflects “DT-Native”, the key features of it, and the running paradigm.

### 2.1. Overview

**Figure 1:**
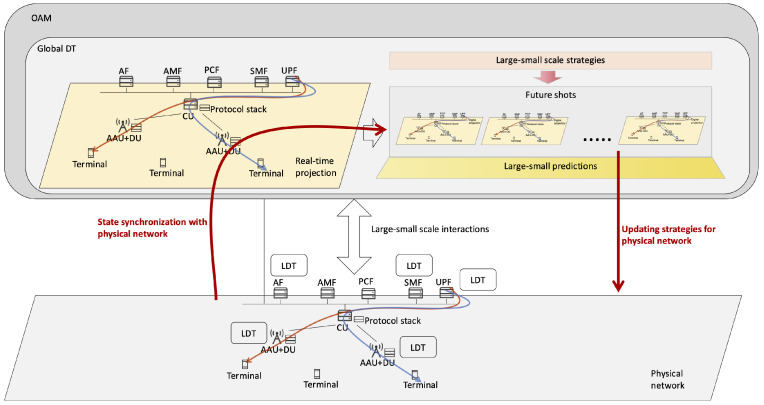
Architecture of DT-native network.

A DT-native network integrates a hierarchical physical network with digital twins (DTs) deployed across different network levels, as illustrated in Figure 1. The DTs are organized in a hybrid centralized-distributed architecture to enable efficient autonomous network management. Specifically, a global DT is deployed to monitor and guide the evolution of the entire physical network, while local DTs (LDTs) are associated with specific physical entities, such as gNBs, terminals, and AMFs, to manage localized areas. This hybrid structure leverages centralized oversight for high-level coordination and distributed processing for real-time, area-specific management [13].

The operation of DTs in a DT-native network relies on their ability to create a virtual space that extends beyond the physical network. Unlike the physical network, which has fixed nodes and edges, the virtual space is dynamic with soft and flexible boundaries. DTs can dynamically add or remove nodes and edges in the virtual space to reflect evolving network requirements. For example, adding a node may involve deploying new physical equipment (via robots or humans) or instantiating software on a cloud-native platform, while deleting a node can be achieved by setting equipment to sleep mode or physically removing it. This flexibility allows DTs to adapt the network structure proactively, supporting both operational maintenance and real-time optimization for network evolution.

To realize these capabilities, each DT incorporates several specialized modules: real-time projection, future shots, autonomous large–small-scale strategy generation (ALSSSG), differentiating long–short-term prediction (DLSTP), and large–small time-scale controls (LSTSCs). The real-time projection module synchronizes the physical network’s states and strategies into the virtual space, providing a foundation for network evolution analysis. The future shots module, driven by DLSTP, projects the physical network’s evolving states, strategies, and performance across multiple time scales under strategies generated by ALSSSG. Finally, the LSTSC module facilitates interactions between the DT virtual space and its physical counterpart, enabling closed-loop control to implement network evolution strategies. Together, these modules ensure that DTs support both immediate network management and long-term self-evolution.

Notice that the DT-native network supports integration with existing network infrastructures and backward compatibility. First, the centralized DT, deployed within the OAM system, mirrors the integration of Self-Organizing Network (SON) functionalities in current networks, leveraging standardized interfaces and algorithms to ensure seamless incorporation into existing OAM frameworks. The primary challenge lies in designing these algorithms and open interfaces, which can utilize established protocols to maintain compatibility with legacy systems. Second, in the distributed deployment, LDTs are embedded within the 6G RAN, which is envisioned to adopt a service-based architecture [13]. This architecture enables network elements, such as gNBs, to be composed of scalable, cloud-native services, allowing the flexible addition of DT services alongside other novel services like sensing. This modularity supports phased deployments, enabling partial integration with 5G RAN elements during the transition to 6G, thereby ensuring backward compatibility and minimizing disruptions to existing infrastructures. The abbreviations of this paper are depicted in Table 1.

### 2.2. Future Shot

The concept of “future shot” is a vital feature of a DT-native network. In a DT, its “future shots” part gives the comprehensive network status of its physical counterpart, which captures the evolving states, strategies, and performance of network entities (e.g., network elements, terminals, traffic). Unlike related concepts in the literature, such as prediction or pre-verification [13,14], which typically focus on isolated variables, “future shots” provides a holistic view of network evolution. Therefore, the “future shots” is a collaborative production of several technologies, such as network prediction, strategy generation, pre-validation, etc. In this way, a DT-native network can realize autonomous network planning, optimization and evolving. There are two key questions to answer:

What time period in future shot: Numerous future shots would lead to enormous cost because of the large network scale and computing workload for predictions. To reduce cost, the predicting time periods of different elements are different, which may change in varying time. On the one hand, part of the elements may be the most possibly problematic part, whose number would increase as the length of time increases, e.g., according to the ages of different types of equipments, the critical or challenging traffics, movements of people, etc. Accordingly, their predicting time period would become smaller as time goes by as well. On the other hand, near future shots are proper to fine-grain, while long future shots are proper to coarse-grained. For example, near future shots can predict the states of network elements or types of resources and give resource optimization results for them for real-time optimization. By comparison, long future shots can predict the network capacity and overall service qualities, and they can also give large-scale network evolution strategies like adding new technologies, e.g., Terahertz, Reconfigurable Intelligent Surface (RIS), etc., and new Active Antenna Unit (AAU) station planning. In addition, the predicting time period of elements in near and long future shots may dynamically changing according to the real condition of the physical network.

What needs to be accomplished in generating future shot: It involves adding results of the predictions of dynamic network self-changes and the performance under autonomous network strategies. First, the basic work is to predict future states of self-changing elements, e.g., user trajectories, traffic distributions, etc. Second, it needs to predict the performance of small-scale optimization strategies and large-scale evolution strategies. If the performance does not achieve the performance requirement, the DT automatically analyzes the bad reason, and then it generates and trains a proper strategy whose performance updates the network condition in future shots. If the ultimate performance still cannot reach the requirement, the DT would inform operators to seek solutions in advance.

In summary, in order to modify the physical network in advance, a DT-native network needs to generate a series of future shots in near and long future time for short–long-scale network evolution.

### 2.3. Hybrid Centralized–Distributed Autonomy

It is appropriate for a physical mobile network with hierarchical features to use hybrid centralized–distributed DTs for autonomous network management with high efficiency [13]. The reason lies in the fact that while it is recommended that OAM platforms deploy centric global DT for high-level management, it is recommended that RANs deploy sub-centralized RAN LDTs for efficient areal management, and network elements deploy distributed LDT for real-time local management.

Concretely, the global DT, hosted on centralized equipment, maintains a comprehensive view through its future shots, which encapsulate two primary types of elements: components critical to future global performance or behavior trends, such as those with significant influence on network-wide dynamics; and components prone to global issues, such as traffic congestion or network overload.

These future shots ensure that the global DT captures non-negligible factors for predicting the impact of network components on overall system behavior.

In contrast, LDTs deployed at individual network elements or regional levels focus on localized future shots that include elements vital to future local performance and potentially problematic entities. For instance, the behavior and performance of high-priority user devices are prioritized in LDT future shots to anticipate local challenges, such as resource contention or service degradation.

The interaction between the global DT and LDTs is orchestrated to optimize network performance and evolution. The global DT evaluates the importance of physical entities to prioritize LDTs, determining the granularity of their predictions and specifying the information they should feed back. This feedback mechanism enables the global DT to guide LDTs in executing optimization or evolution strategies. For example, when the global DT predicts potential cell congestion in a specific region, it instructs all LDTs in that region to perform fine-grained traffic predictions over a specified future time window. Based on the aggregated LDT predictions, the global DT coordinates the configuration of cell sleep strategies, which is a task that LDTs cannot perform independently due to their localized perspective. This collaborative process ensures that local optimizations align with global network objectives, facilitating efficient and autonomous network evolution.

It needs to be mentioned that in order to realize hierarchical controls, DTs of different layers may use different technologies, which may be intertwined with each other’s performance. For example, cell-free technology is an RAN-level technology, which may influence RIS (for single gNB) performance.

## 3. Key Technologies of DT-Native Network

In DT-native networks, to realize network self-evolution, efficient DLSTP and ALSSSG systems need to be designed to support future shots, and proper LSTSC needs to be proposed to accomplish physical evolution. Therefore, this section sequentially gives general methods for DLSTP, ALSSSG and LSTSC to facilitate self-evolution in 6G networks.

### 3.1. Differentiating Long–Short-Term Prediction

In a DT-native network, DLSTP needs to predict the future states and strategy performance (also called pre-validation) for different network elements. The states and performance can be taken as properties of network elements; e.g., the properties of a gNB can include the number of access terminals (states) and cell capacity (performance). The predicting process can be split into two steps: first giving the predicting strategy (including the predicting time period and predicting time length) for different elements and then giving predicting results. The details are as follows.

#### 3.1.1. Determining Predicting Strategies

Issue: DLSTP needs to determine the potentially problematic network elements that when combined may place excessive demand on the system performance and computing or memory cost. These may include fluctuating elements, like the mobile device trajectory, services traffic, and equipment default probability, as well as performance metrics like the network capacity and workload distribution on different network elements. Considering that the properties of different elements interact with each other, DLSTP needs to jointly consider different elements to give predicting strategies. The strategy needs to include the predicting time period and predicting time-length.

Solution: To generate a proper predicting strategy, we propose a cross-time hierarchical graph attention model (CTHGAM), which is denoted as $S_{O,\mathrm{CTHGAM}}$. The input is the predicting results for the future, with different predicting time periods and predicting time numbers (their product result is the predicting time length). Based on this, the model needs to decide whether any element performance is decreasing, which need to give a smaller predicting time period and larger predicting time number. Meanwhile, it needs to decide whether an element performance influences other critical performance, which can be trained to learn via a predicting loss function. The output gives the predicting time period and predicting time number, suggested for the next predicting decision period, for different elements. The details are as follows:

Firstly, entities are grouped based on hierarchical relationships, where entities from different network layers are allocated into different groups. For example, globally connected components (e.g., AMF, UPF, CU) are allocated into one group, while DUs under different CUs and user devices connected to different AAUs are assigned separate groups. Each group is denoted as $\Psi_{g}\;\left(g\in G\right)$, where G={1,2,…,G}.

**Figure 2:**
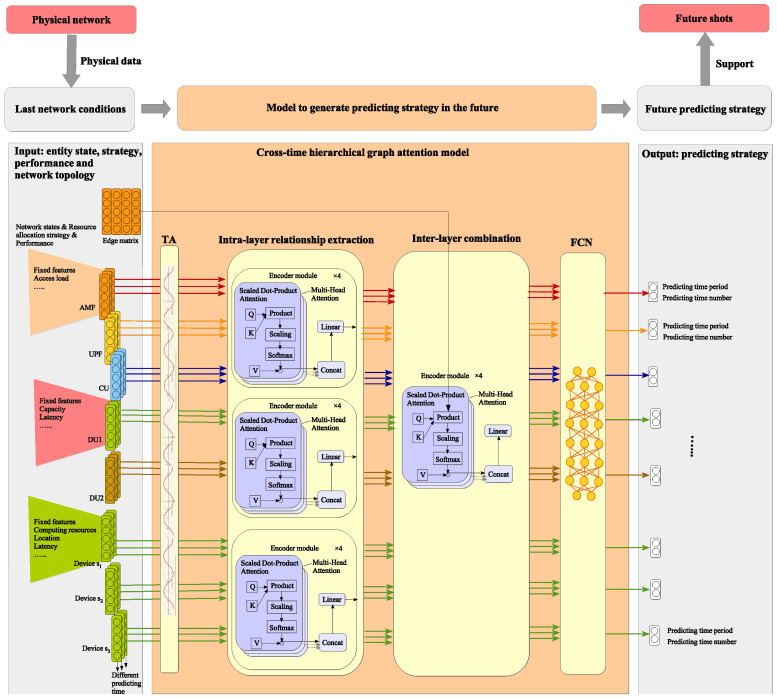
Proposed cross-time hierarchical graph attention model (CTHGAM).

Secondly, the model inputs the states, performances, strategies, and previous predicting time periods of different network elements from different groups as well as the hierarchical network topology for generating a predicting strategy. The architecture of the CTHGAM can be expressed as

(1) $$\begin{array}{cc} \; & S_{O,\mathrm{CTHGAM}}\left(S_{n\in \Psi },\zeta_{n\in \Psi },P_{n\in \Psi },U,\tau_{n\in \Psi }^{\mathrm{now}}\right)=\\ \; & \mathrm{FCN}\left(\mathrm{GphAttention}\left(\left\{{\mathrm{MultiHead}}_{g}\left(\mathrm{TA}\left(\left(S_{n,i-1},P_{n,i-1},\zeta_{n,i-1}\right),\tau_{n,i-1}^{\mathrm{now}}\right){|}_{i=1,\ldots ,I;n\in \Psi_{g}}\right)\right\}{|}_{g\in G},U\right)\right) \end{array}$$

where $S_{n}$ gives the state set of the *n*-th physical element, $\zeta_{n}$ is the set of network strategies on the *n*-th physical element, $P_{n}$ is its set of performance metrics, U is the network topology, $\tau_{n}^{\mathrm{now}}$ is the time step of the *n*-th physical element, (i−1) represents the latest prediction iteration, $\tau_{n,i-1}^{\mathrm{now}}$ gives the time step of latest predicting result of the *n*-th physical element, and model parts TA(·), ${\mathrm{MultiHead}}_{g}\left(\cdot \right)\;\left(g\in G\right)$, GphAttention(·), and FCN(·) give the operation of different layers. In detail, the model comprises four layers, where the first layer comprises a time alignment part TA(·); the second layer comprises a few identical model parts ${\mathrm{MultiHead}}_{g}\left(\cdot \right)\;\left(g\in G\right)$ for intra-group feature extractions; the third layer comprises the model part GphAttention(·) for extracting the inter-group features; and the fourth layer comprises a fully connected network (FCN) for feature integration to generate inferring results. The detail of the whole architecture is depicted in Figure 2. The detail of each layer is designed as follows.

In the first layer, time alignment TA(·) aims at adding different time steps $\tau_{n,i-1}^{\mathrm{now}}$ onto each element input vector $\left(S_{n,i-1},P_{n,i-1},\zeta_{n,i-1}\right)$. It is designed using the expression of positional encoding based on sine and cosine functions as given in Appendix A.

The second and third layer collaboratively form a hierarchical graph attention model. It can dynamically weigh the importance of different topological connections within and across different network layers. This allows the CTHGAM model to focus on the most relevant entities and their interactions when giving the predicting strategy of a given network element.

In detail, in the second layer, network elements from different groups transmit their states, strategies, and performance to ${\mathrm{MultiHead}}_{g}\left(\cdot \right)$, which captures entity features and inter-group relationships using an attention-based encoder. Considering lightweight models are preferred to efficiently extract feature relationships among network entities, the parameters of the attention module are designed to be lighter than those typically used in traditional LLMs. The number of attention heads can be set to 4, with four encoder modules. The whole process is expressed as follows

(2) $$\begin{array}{c} a_{1}^{\left(3\right)},a_{2}^{\left(3\right)},\ldots ,a_{d\times n_{G_{g}}}^{\left(3\right)}={\mathrm{MultiHead}}_{g}\left(\mathrm{TA}\left(\left(S_{n,i-1},P_{n,i-1},\zeta_{n,i-1}\right),\tau_{n,i-1}^{\mathrm{now}}\right){|}_{i=1,\ldots ,I;n\in \Psi_{g}}\right) \end{array}$$

where $n_{G_{g}}$ denotes the number of entities in the *g*-th group, and $a^{\left(3\right)}$ is the second-layer output. Thus, each entity’s property vector in $a^{\left(3\right)}$ integrates its original features with both known and unknown inter-group relationships. For instance, CU-related vectors capture positions and connections with AMF and UPF, DU-related vectors reflect resource and user access dynamics, and user device-related vectors learn data distribution and wireless environment interactions within the same AAU.

In the third layer, outputs from different groups pass through a graph attention module, which computes *Q* and *K* matrices and applies hierarchical topology. The module’s architecture is as follows

(3) $$\begin{array}{c} \mathrm{GphAttention}\left(\cdot \right)=\mathrm{softmax}\left(\frac{QK^{T}U}{\sqrt{d}}\right)V \end{array}$$

where *V* is the value matrix. The third layer processes vectors from different element groups as follows

(4) $$\begin{array}{c} a_{1}^{\left(4\right)},...,a_{d\times n_{G_{1}}+d\times n_{G_{2}}+d\times n_{G_{3}}}^{\left(4\right)}=\mathrm{GphAttention}\left(\left\{a_{1}^{\left(3\right)},a_{2}^{\left(3\right)},...,a_{d\times n_{G_{g}}}^{\left(3\right)}\right\}{|}_{g\in G},U\right) \end{array}$$

By incorporating GphAttention(·), the third layer integrates features of groups with direct relationships, such as user devices and their associated AAU. Additionally, multiple repetitions of GphAttention(·) enable the integration of weakly related groups, offering supplementary references for the final learning objective.

The fourth layer is a feature integration layer that merges various element features. Its output represents the global intelligent model’s inference goals, such as latency and capacity estimations. Aligned with the FCN architecture, it is expressed as follows

(5) $$\begin{array}{c} {\left[{\mathrm{PD}}_{\mathrm{len},n},{\mathrm{PD}}_{\mathrm{num},n}\right]}_{n\in \Psi }=\mathrm{FCN}\left(a_{1}^{\left(4\right)},...,a_{d\times n_{G_{1}}+d\times n_{G_{2}}+d\times n_{G_{3}}}^{\left(4\right)}\right) \end{array}$$

where ${\mathrm{PD}}_{\mathrm{len},n}and{\mathrm{PD}}_{\mathrm{num},n}$ are the predicting period and predicting time number of the *n*-th element. The algorithm step of CTHGAM is depicted in Algorithm 1.
**Algorithm 1** Cross-Time Hierarchical Graph Attention Model (CTHGAM)  1:**Input:** Network element states $S_{n,i-1}$, performance metrics $F_{n,i-1}$, strategies $\zeta_{n,i-1}$, previous time straps $\tau_{n,i-1}^{\mathrm{now}}$, network topology U, groups $\Psi_{g}\left(g\in G\right)$  2:**Output**: Predicting period ${\mathrm{PD}}_{\mathrm{len},n}$, predicting time number ${\mathrm{PD}}_{\mathrm{num},n}$ for each element n∈Y  3:* Layer 1: Time Alignment*  4:**for** each element n∈Y **do**  5:    Compute time-aligned features using positional encoding:  6:    $\mathrm{TA}\left(\left(S_{n,i-1},F_{n,i-1},\zeta_{n,i-1}\right),\tau_{n,i-1}^{\mathrm{now}}\right)\leftarrow \mathrm{FCN}\left(S_{n,i-1},F_{n,i-1},\zeta_{n,i-1}\right)+{\left\{\sin\left(\tau_{n,i-1}^{\mathrm{now}}/10000^{2j/d}\right)\right\}}_{j=1,\ldots ,d}$  7:    $\mathrm{TA}\left(\left(S_{n,i-1},F_{n,i-1},\zeta_{n,i-1}\right),\tau_{n,i-1}^{\mathrm{now}}\right)\leftarrow \mathrm{FCN}\left(S_{n,i-1},F_{n,i-1},\zeta_{n,i-1}\right)+{\left\{\cos\left(\tau_{n,i-1}^{\mathrm{now}}/10000^{2j/d}\right)\right\}}_{j=1,\ldots ,d}$  8:**end for**  9:* Layer 2: Intra-Group Feature Extraction*10:**for** each group g∈G **do**11:    Extract intra-group features using multi-head attention:12:    $\left\{a_{1}^{\left(3\right)},a_{2}^{\left(3\right)},\ldots ,a_{d\times n_{C_{g}}}^{\left(3\right)}\right\}\leftarrow {\mathrm{MultiHead}}_{g}\left({\left\{\mathrm{TA}\left(\left(S_{n,i-1},F_{n,i-1},\zeta_{n,i-1}\right),\tau_{n,i-1}^{\mathrm{now}}\right)\right\}}_{n\in \Psi_{g}}\right)$13:**end for**14:* Layer 3: Inter-Group Feature Extraction*15:Compute inter-group features using graph attention:16:$\left\{a_{1}^{\left(4\right)},\ldots ,a_{d\times n_{C_{1}}+d\times n_{C_{2}}+\ldots }^{\left(4\right)}\right\}\leftarrow \mathrm{GphAttention}\left({\left\{a_{1}^{\left(3\right)},a_{2}^{\left(3\right)},\ldots ,a_{d\times n_{C_{g}}}^{\left(3\right)}\right\}}_{g\in G},U\right)$17:$\mathrm{GphAttention}\left(\cdot \right)=\mathrm{softmax}\left(\frac{QK^{T}U}{\sqrt{d}}\right)V$18:* % Layer 4: Feature Integration*19:Integrate features to generate predicting strategies:20:${\left[{\mathrm{PD}}_{\mathrm{len},n},{\mathrm{PD}}_{\mathrm{num},n}\right]}_{n\in Y}\leftarrow \mathrm{FCN}\left(a_{1}^{\left(4\right)},\ldots ,a_{d\times n_{C_{1}}+d\times n_{C_{2}}+\ldots }^{\left(4\right)}\right)$21:**Return** ${\mathrm{PD}}_{\mathrm{len},n},{\mathrm{PD}}_{\mathrm{num},n}$ for all n∈Y

#### 3.1.2. Giving Predicting Results

Issue: After obtaining a proper predicting strategy, the next step for DLSTP is to generate predicting results for different network elements based on the predicting strategy. The predicting result for each element needs to jointly consider its own previous states and the co-influence of different elements under an acceptable cost.

**Figure 3:**
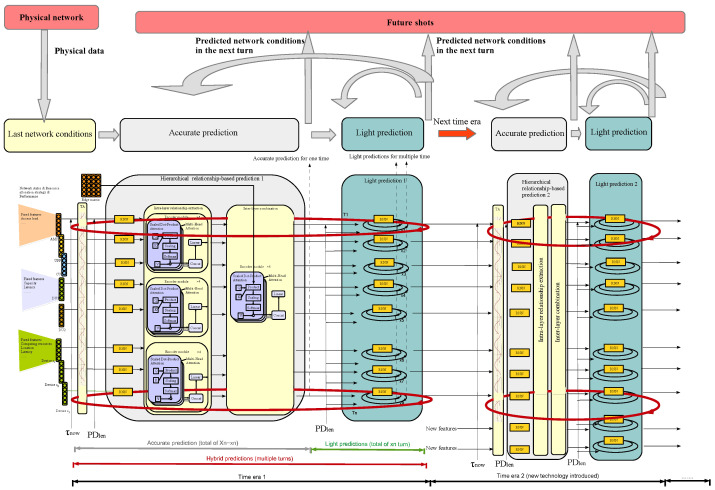
The process of proposed long-term hierarchical convolutional graph attention model (LTHCGAM).

Solution: To efficiently generate an accurate predicting result, we propose a long-term hierarchical convolutional graph attention model (LTHCGAM). In detail, future times are divided into different time eras, which indicates the network gradually evolves into different eras, e.g., by introducing new technologies or accomplishing new network planning solutions. In each time era, predictions for different elements are iterated between accurate prediction and light predictions (i.e., a hybrid predicting process), where the predicted time period and predicted time number are determined by the proposed predicting strategy and are different between elements. The predicting results would be integrated into different future shots, where the coarsely predicted results remain stable over a small-time scale so as to align the time periods between different elements. When a time era is over, the last predicting results of it are sending to the next hybrid prediction module designed for the next era. Different hybrid prediction modules between time eras are similar but with different sizes to adapt to different numbers of features in different eras (e.g., new technology introduced). The process is depicted in Figure 3.

The overall algorithm can be expressed as follows

(6) $$\begin{array}{cc} \; & S_{n,i},P_{n,i},\zeta_{n,i}=S_{O,\mathrm{CTHGAM}}\left(S_{n\in \Psi },P_{n\in \Psi },U,\zeta_{n\in \Psi },\tau_{n\in \Psi }^{\mathrm{now}}\right)=\\ \; & \left\{\begin{array}{c} \mathrm{GphAttention}\left(\left\{{\mathrm{MultiHead}}_{g}\left(\mathrm{RNN}\left(\mathrm{TA}\left(\left(S_{n,i-1},P_{n,i-1},\zeta_{n,i-1}\right),\tau_{n}^{\mathrm{now}}\right),{\mathrm{PD}}_{\mathrm{len},n}\right){|}_{n\in \Psi_{g}}\right)\right\}{|}_{g\in G},U\right),\;\mathrm{if}i=I_{\gamma }^{\mathrm{start}}+1+\xi_{n,\gamma }k,\\ \mathrm{RNN}\left(S_{n,i-1},P_{n,i-1},\zeta_{n,i-1},{\mathrm{PD}}_{\mathrm{len},n}\right),\;\mathrm{else} \end{array}\right. \end{array}$$

where time era γ is determined based on $i\cdot L_{n}$, and $I_{\gamma }^{\mathrm{start}}$ is the iteration number of time era γ. In a time era, the predicting procedure iteratively goes through one accurate predicting process (corresponding to the first condition) and multiple light but less accurate predicting processes (corresponding to the second and third conditions). Next, the details of the two processes are sequentially given as follows.

As for the accurate predicting process, first, predictions are input to time alignment TA(·) to align the predicting time period for different network elements as follows

(7) $$\begin{array}{cc} \; & \mathrm{TA}\left(\left(S_{n,i-1},P_{n,i-1},\zeta_{n,i-1}\right),\tau_{n}^{\mathrm{now}}\right)=\mathrm{FCN}\left(S_{n,i-1},P_{n,i-1},\zeta_{n,i-1}\right)+\left\{\sin(\tau_{n}^{\mathrm{now}}/10,000^{2j/d})\right\}{|}_{j=1,\ldots ,d}\\ \; & \mathrm{TA}\left(\left(S_{n,i-1},P_{n,i-1},\zeta_{n,i-1}\right),\tau_{n}^{\mathrm{now}}\right)=\mathrm{FCN}\left(S_{n,i-1},P_{n,i-1},\zeta_{n,i-1}\right)+\left\{\cos(\tau_{n}^{\mathrm{now}}/10,000^{2j/d})\right\}{|}_{j=1,\ldots ,d} \end{array}$$

which aims at adding different time steps $\tau_{n}^{\mathrm{now}}$ onto each element input vector $\left(S_{n,i-1},P_{n,i-1},\zeta_{n,i-1}\right)$. Then, it inputs to a hierarchical relationship-based prediction module for accurate prediction.

(8) $$\begin{array}{c} S_{n,i},P_{n,i},\zeta_{n,i}=\mathrm{GphAttention}\left(\left\{{\mathrm{MultiHead}}_{g}\left(\mathrm{RNN}\left(\mathrm{TA}\left(\left(S_{n,i-1},P_{n,i-1},\zeta_{n,i-1}\right),\tau_{n}^{\mathrm{now}}\right),{\mathrm{PD}}_{\mathrm{len},n}\right){|}_{n\in \Psi_{g}}\right)\right\}{|}_{g\in G},U\right) \end{array}$$

where RNN(·) dictates the historical state of each element, while MultiHead(·) and GphAttention(·) form a graph attention model similar to the one in Section 3.1.1, which allows the model to focus on the most relevant entities and their interactions when predicting the future state of a given network element on its next predicting time in $\tau_{n}^{\mathrm{now}}$.

As for the light predicting process, the predicting result of the next predicting turn for each element is generated only based on its own historical data (more accurately the latest predicting data), which is expressed by

(9) $$\begin{array}{c} S_{n,i},P_{n,i},\zeta_{n,i}=\mathrm{RNN}\left(S_{n,i-1},P_{n,i-1},\zeta_{n,i-1},{\mathrm{PD}}_{\mathrm{len},n}\right) \end{array}$$

Note that we give an RL-based approach to dynamically determine the number of cycles for accurate prediction $\left(\chi_{n}-x_{n}\right)$ and lightweight predictions $x_{n}$ under a fixed total number of prediction rounds $\chi_{n}$. The RL framework balances prediction accuracy and computational complexity by defining a reward function as follows:

(10) $$\begin{array}{c} R=w_{1}\cdot ∥\vec{\left(S_{n,i}-{\hat{S}}_{n,i},P_{n,i}-{\hat{P}}_{n,i},\zeta_{n,i}-{\hat{\zeta }}_{n,i}\right)}∥-w_{2}\cdot \left\{C_{\mathrm{AP}}\left(\chi_{n}-x_{n}\right)+x_{n}C_{\mathrm{LP}}\right\} \end{array}$$

where $w_{1}$ and $w_{2}$ are weighting factors, $S_{n,i}$, ${\hat{S}}_{n,i}$, $P_{n,i}$, ${\hat{P}}_{n,i},\zeta_{n,i}$, and ${\hat{\zeta }}_{n,i}$ are the predicted and real network states, performance and strategies, respectively, and $C_{\mathrm{AP}}$ and $C_{\mathrm{LP}}$ are the computing complexity of accurate prediction and lightweight prediction, respectively. After each prediction cycle, physical data are collected to compute the reward, and the cycle counts $\left(\chi_{n}-x_{n}\right)$ for precise and cycle counts $x_{n}$ for lightweight predictions are updated online. This adaptive approach ensures that the model continuously adjusts to environmental changes in the mobile network, maintaining an optimal balance between accuracy and efficiency. The algorithm step of LTHCGAM is depicted in Algorithm 2.
**Algorithm 2** Long-Term Hierarchical Convolutional Graph Attention Model (LTHCGAM)  1:**Input:** Network element states $S_{n,i-1}$, performance metrics $F_{n,i-1}$, strategies $\zeta_{n,i-1}$, predicting strategy ${\mathrm{PD}}_{\mathrm{len},n}$, ${\mathrm{PD}}_{\mathrm{num},n}$, topology U, groups $\Psi_{g}\left(g\in G\right)$, time eras γ∈Γ, total prediction rounds $\chi_{n}$  2:**Output:** Predicted states $S_{n,i}$, performance $F_{n,i}$, strategies $\zeta_{n,i}$ for each element n∈Y  3:Initialize RL parameters: weights $w_{1}$, $w_{2}$, accurate prediction cost $C_{\mathrm{AP}}$, lightweight prediction cost $C_{\mathrm{LP}}$  4:**for** each time era γ∈Γ **do**  5:    Determine iteration start $l_{\gamma }^{\mathrm{start}}$ based on $i\cdot L_{n}$  6:    Initialize hybrid prediction module for era γ with adaptive feature sizes  7:    Compute cycle counts for accurate ($\chi_{n}-x_{n}$) and lightweight ($x_{n}$) predictions using RL:  8:    $R=w_{1}\cdot ∥\left({\bar{S}}_{n,i}-{\hat{\bar{S}}}_{n,i},{\bar{F}}_{n,i}-{\hat{\bar{F}}}_{n,i},{\bar{\zeta }}_{n,i}-{\hat{\zeta }}_{n,i}\right)∥-w_{2}\cdot \left\{C_{\mathrm{AP}}\left(\chi_{n}-x_{n}\right)+x_{n}C_{\mathrm{LP}}\right\}$  9:    **for** each iteration *i* in era γ **do**10:        **if** accurate prediction condition **then**11:           *Accurate Prediction*12:           Align time steps:13:           $\mathrm{TA}\left(\cdot \right)\leftarrow \mathrm{FCN}\left(S_{n,i-1},F_{n,i-1},\zeta_{n,i-1}\right)+{\left\{\sin\left(\tau_{n}^{\mathrm{now}}/10000^{2j/d}\right)\right\}}_{j=1,\ldots ,d}$14:           $\mathrm{TA}\left(\cdot \right)\leftarrow \mathrm{FCN}\left(S_{n,i-1},F_{n,i-1},\zeta_{n,i-1}\right)+{\left\{\cos\left(\tau_{n}^{\mathrm{now}}/10000^{2j/d}\right)\right\}}_{j=1,\ldots ,d}$15:           Record historical states: $\mathrm{RNN}(\mathrm{TA}\left(\cdot \right),{\mathrm{PD}}_{\mathrm{len},n})$16:           Extract features:17:           $S_{n,i},F_{n,i},\zeta_{n,i}\leftarrow \mathrm{GphAttention}\left({\left\{{\mathrm{MultiHead}}_{g}{\left(\mathrm{RNN}(\mathrm{TA}\left(\cdot \right),{\mathrm{PD}}_{\mathrm{len},n})\right)}_{n\in \Psi_{g}}\right\}}_{g\in G},U\right)$18:        **else**19:           *Lightweight Prediction*20:           Predict based on historical data:21:           $S_{n,i},F_{n,i},\zeta_{n,i}\leftarrow \mathrm{RNN}\left(S_{n,i-1},F_{n,i-1},\zeta_{n,i-1},{\mathrm{PD}}_{\mathrm{len},n}\right)$22:        **end if**23:        Collect physical data and update RL reward *R*24:        Update cycle counts $\chi_{n}-x_{n}$, $x_{n}$ based on *R*25:    **end for**26:    Pass final predictions to next era’s hybrid prediction module27:**end for**28:Integrate predictions into future shots, aligning time periods29:**Return** $S_{n,i},F_{n,i},\zeta_{n,i}$ for all n∈Y

### 3.2. Autonomous Large–Small-Scale Strategy Generation

The “future shot” comprehensively demonstrates the network state, network strategies, and network performance across different times in a future network. If network performance is suboptimal during a certain period, it becomes necessary to automatically generate improved network strategies to enhance performance. Here, network strategies encompass strategies of “large and small scales”. The word “scale” has two meanings, including the “scope” of its application and “magnitude” of the improvement approach. The former indicates whether it pertains to the entire network range managed by an OAM platform, a specific local area within a RAN, or an individual network element node. The latter indicates whether it involves optimizing configuration parameters based on existing technology or making significant adjustments by adopting or introducing alternative technologies. The following provides a detailed description of the strategy generation process.

**Figure 4:**
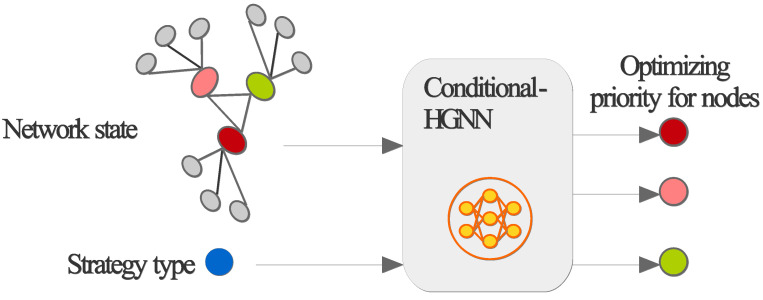
Generating optimizing priority for nodes using Conditional-HGNN.

First, demand self-awareness is required based on the results of “future shots” to identify the time point when performance begins to degrade, determine which technology is needed to address it, and pinpoint the critical network element node for resolving the degradation. This is categorized into two scenarios based on the scope of application:

- Large-scope self-awareness: For example, it may be demand self-awareness on an OAM platform, which can be implemented in three steps. First, it can identify a time point after which performance falls below a certain threshold. Then, it can use an LLM model to provide recommendations on which technology should be employed for optimization or evolution. The LLM’s foundation model can be trained on extensive datasets comprising historical network data, laboratory data, and standardized communication protocols, which had been used by researchers to develop network large models [15]. The LLM can be further fine-tuned using supervised learning on labeled datasets derived from historical network optimization scenarios and reinforcement learning with a reward function that prioritizes KPIs such as reduced latency, increased throughput, and enhanced reliability, ensuring alignment with the network’s self-evolution objectives. Finally, the heterogeneous graph, formed by the heterogeneous performance of various network elements with their connecting topology, and the optimization method suggested by the LLM are input into a conditional-heterogeneous graph neural network (Conditional-HGNN) to output a prioritized ranking of network nodes recommended for optimization, as depicted in Figure 4.
- Small-scope self-awareness: For example, it may be demand self-awareness on a single network element, which is implemented in two steps. First, it can identify a point in time after which performance falls below a certain threshold. Then, it uses an LLM to suggest which technology should be used for optimization or evolution.

Second, generate the initial state of the specific strategy, train the final configuration parameters, and deploy them to “future shots”. In mobile communication networks, the optimization approaches can be divided into two types: small-magnitude optimizations and large-magnitude optimizations. Concretely, small-magnitude optimizations involve incremental refinements of existing parameters, such as antenna weights or resource scheduling, to enhance performance within the current architecture with lower complexity. In contrast, large-magnitude optimizations introduce transformative technologies, like cell-free architectures or integrated sensing and communication, to address fundamental limitations, requiring significant infrastructural changes. This classification reflects the differing scopes and impacts of these strategies. Based on this consideration, we give their separate approaches as follows:

- Large magnitude: If the improvement approach is significant and requires introducing new technology, it involves two steps. First, it initializes configuration parameters for the new technology. If a flexible, intelligent, and dynamic configuration scheme based on AI is needed, an LLM can be used to define the AI model’s structure and initial parameters. If AI-based configuration is not required, a preset template can be used for initialization. Second, it performs the adaptive optimization of the initial configuration. Concretely, it can build a performance pre-validation environment based on the APIs of different network elements to train AI-based configuration strategies or optimize non-AI configuration schemes based on templates.
- Small magnitude: If the improvement approach is minor and only requires optimizing the configuration parameters of existing technology, proceed directly with the second step above.

Note that if a “future shot” undergoes strategy optimization, subsequent “future shots” predictions must be adjusted accordingly. If the ultimate performance still cannot reach the requirement, the DT would inform operators to seek solutions in advance, which would be dictated into a database for suggesting an optimizing strategy in the future.

### 3.3. Large–Small Time-Scale Controls

A DTN manages the physical network across large and small time scales, leveraging the network strategies and performance data from “future shots” to optimize and evolve the physical network in a layered manner. The concept of “scale” encompasses two key aspects.

Optimization and Evolution: An NDT’s autonomous control over the physical network often involves simultaneous small-scale optimization and large-scale evolution.

- On a small time scale, an NDT quickly interacts with the physical network to continuously optimize the physical network. By deploying distributed NDT modules on its physical counterpart, it can timely update network AI algorithms via inner physical–virtual interfaces to quickly adapt to the changing physical environments, ensuring precise and efficient strategies for resource allocation, mobility management, and other network operational states.
- On a large time scale, an NDT periodically interacts with its physical network to facilitate its gradual evolution. Many network evolution strategies require long-term, incremental progression and directional transitions. For instance, a cell-free network might begin with pilot deployments in areas with fewer users and then progressively expand to densely populated regions until the entire network is fully upgraded. By deploying a central NDT module on an OAM, CU or other centralized operation places, it can have an overview of the whole network and gradually emit controls over it on a large time scale.

Local and Global: An NDT employs a centralized–distributed deployment architecture, where local control is suited for smaller time-scale operations, and global control is tailored to larger time-scale management. This requires cost-effective, multi-time-scale top–down control.

- At the smallest time scale, an network element DT is deployed alongside its physical counterpart, performing real-time status monitoring and closed-loop control of the equipment.
- At the largest time scale, a DT is deployed within an operation and maintenance administration system, enabling large time-scale optimization and control across the entire network.

It needs to be mentioned that effective “large–small” time-scale control is only achievable by simultaneously addressing both “local and global” and “optimization and evolution”. For example, within the DT of a single network element under “small time scale control”, there may be two distinct time-scale controls: optimization and evolution. Take the DT of a gNB as an example—it not only needs to handle network optimization controls like resource allocation but also evaluate whether to introduce new technologies such as terahertz (THz) or reconfigurable intelligent surfaces (RISs) for iterative network evolution.

## 4. Use Cases

This section presents various application scenarios of DT-native networks, encompassing large language models and AGI, transportation and driving, industrial internet, and low altitude airspace economy.

### 4.1. Large Language Models and AGI

With the continuous advancement of network intelligence, LLMs and Artificial General Intelligence (AGI) are being introduced into network operation and maintenance to address challenges such as the complexity of network management, achieving notable positive outcomes in some cases [19]. However, due to difficulties in obtaining network data and inconsistent data quality, LLMs and AGI may exhibit hallucination phenomena and produce inaccurate inferences and perception, thereby adversely affecting network performance or even causing systemic failures. Therefore, the pre-validation of intelligent decision feasibility is a critical step to ensure stable network operations.

In a DT-native network, the NDT, as a high-fidelity digital replica of the physical network, plays a vital role in addressing these challenges. Firstly, an NDT can simulate arbitrary network states to generate large volumes of high-quality and training data for LLMs, effectively solving the generalization limitations. LLMs often struggle to adapt to diverse scenarios, especially unexpected or rare network events, due to a lack of diverse training datasets. By leveraging NDTs, these models can train on simulated data that include complex and abnormal network states, thereby significantly enhancing their adaptability and generalization performance. At the same time, NDTs provide interactive and dynamic data specifically for AGI, enabling it to refine its understanding of complex network behaviors and improve its decision-making processes in a safe, controlled environment. This interactive capability also contributes to enhancing the cognitive level of AGI, making it more robust and effective in handling real-world network operations. Secondly, an NDT serves as an interactive training environment for LLM and AGI learning. It allows these models to interact with simulated network environments, replacing risky direct interactions with physical networks. During training, the feedback provided by NDTs, such as performance metrics or simulated network responses, acts as reinforcement signals, helping LLMs continuously optimize their strategies and achieve faster convergence and improving AGI cognitive level. Lastly, an NDT provides a critical validation platform for intelligent decisions generated by LLMs or AGI. Before deploying these decisions in physical networks, an NDT allows for pre-configuration and simulation of their execution. If a decision proves to be infeasible during simulation, the model iterates further until an optimal and feasible strategy is achieved. Only then is the decision deployed to the physical network, ensuring minimal risk of performance degradation or network failure caused by erroneous decisions. This closed- loop validation process not only enhances the reliability of intelligent decision making but also builds trust in the deployment of LLMs and AGI across various network scenarios.

In summary, NDTs provide interactive training environments and validate intelligent decisions, making them an indispensable tool for applying LLMs and AGI into network intelligence. By addressing key challenges such as data quality, model generalization, and decision feasibility, NDTs significantly accelerate the development and deployment of network intelligence, paving the way for more robust and trustworthy AI-driven network systems.

### 4.2. Transportation and Driving

Currently, the intelligent transportation and driving fields face numerous challenges, which are primarily manifested in the complexity of traffic scenarios. A traffic scenario is a multi-element system comprising interconnected components such as people, vehicles, roads, and environments. The construction of current hybrid traffic scenarios presents two main issues: insufficient consideration of the impact of the evolution road dynamics regarding mixed traffic and a lack of multi-person hybrid traffic perception methods.

Traditional on-site testing has significant limitations, struggling to reproduce extreme scenarios and comprehensively simulate complex factors like obstacle volume, angles, vehicle speed, lighting, weather, and sensor conditions with certain hazardous scenarios (such as collisions or rollovers) being nearly impossible to directly collect data from. Actual data collection faces challenges including sample imbalance, scarcity of long-tail and sensitive high-safety domain data, and increasing difficulty in acquiring effective data as algorithm maturity increases.

A DT-native network provides innovative solutions for autonomous driving testing, enabling the high-precision reconstruction of traffic scenarios, simulating real natural environments (rain, snow, lighting), offering more diverse and balanced test scenarios, generating synthetic data to fill gaps in real data, and enhancing data diversity, completeness, and balance. Compared to traditional mileage testing, simulation testing is more economically efficient, capable of covering the most scenarios with the least mileage and shortest time, significantly improving testing efficiency. By integrating maps and digital twin technology, more realistic and accurate simulation environments can be created, mimicking real-world roads, traffic flows, signals, and signs, comprehensively conducting functional, performance, and safety tests, and enhancing user experience.

As technological capabilities continue to be released and implemented, DT-native networks will accelerate the arrival of Level 3 autonomous driving, improve human– machine collaboration capabilities, and realize better human– machine interaction and co-driving.

### 4.3. Industrial Internet

The DT-native network, as an emerging technology, demonstrates enormous potential in the industrial Internet domain. By creating virtual replicas of physical entities, they enable the real-time monitoring, analysis, and optimization of physical systems, thereby improving production efficiency, reducing costs, and enhancing product quality. In the industrial Internet, a DT-native network primarily covers five core application scenarios: equipment management and maintenance, production process optimization, product design and development, supply chain management, and safety production.

In equipment management and maintenance, NDTs collect real-time equipment operational data through sensors, proactively identifying potential failures and predicting equipment failure times based on historical data and machine learning models, thus achieving predictive maintenance. Simultaneously, by simulating equipment operation in virtual environments, they can effectively validate new maintenance proposals and reduce actual operational risks.

In production process optimization, NDTs can simulate the impact of different production parameters on product quality and efficiency, precisely locate production bottlenecks, and rapidly respond to market demand changes, enabling flexible production. In product design and development, they support creating DT models for virtual testing and verification, significantly shortening product development cycles and promoting collaborative design across multiple teams. Within supply chain management, digital twin networks provide end-to-end visualization tracking, improve supply chain transparency, and optimize inventory management by predicting future demands based on data. In terms of safety production, they can identify potential risks, simulate accident scenarios, develop targeted emergency plans, and enhance emergency response capabilities.

The core advantages of a DT-native network in the industrial Internet include significantly improving production efficiency, reducing operational costs, enhancing product quality consistency, strengthening data-driven decision-making capabilities, and creating favorable conditions for the research, development, and application of new technologies and products. This demonstrates their immense potential and strategic value as a key enabling technology in the industrial Internet.

### 4.4. Low Altitude Airspace Economy

One of the main challenges faced by the low-altitude economy is how to rationally allocate network resources to ensure effective scheduling [20]. The low-altitude economic scenario is complex and dynamic, involving various types of UAVs, different task requirements, and constantly changing environmental factors such as weather and terrain. In particular, when multiple UAVs are working together, the dynamic changes in communication and computing resources make resource allocation and flight scheduling even more difficult. Traditional experience-based decision-making methods can no longer meet the rapidly changing demands, particularly in key tasks such as collision avoidance and airspace sharing, which may lead to resource wastage or flight safety risks.

To address these challenges, NDTs provide significant support for the low-altitude economy by advanced simulation and intelligent resource scheduling. Firstly, NDTs can offer high-precision geographic information for the low-altitude economy through accurate low-altitude planning digital maps and sensing data. This information, through the grid space constructed by the NDT, transforms airspace management from continuous trajectory solving to discrete digital grid space probability prediction and control calculation, which plays a crucial role in flight path planning and collision avoidance. Secondly, NDTs can reduce the dimensionality of large-scale traffic flow management optimization problems by employing grid-based traffic flow models and digital grid space relationship decoupling methods, thereby improving decision-making efficiency. Thirdly, NDTs provide an interactive training environment, allowing different types of UAVs to interact in real time in a simulated environment. Based on feedback, UAVs can optimize their task execution and resource scheduling strategies. Through this dynamic optimization process, NDTs can provide real-time feedback on changes in the simulation environment, helping intelligence engines optimize decision strategies based on new information while ensuring personalized communication, computation, and AI service support. This effectively reduces collision probabilities, energy consumption, and improves overall system service quality.

In conclusion, NDTs offer significant advantages for the low-altitude economy by improving resource utilization efficiency and ensuring the safety and sustainable operation of UAV systems. Additionally, NDTs enable the efficient organization of low-altitude resources, driving the intelligent development of the low-altitude economy.

## 5. Conclusions

This paper designs a new architecture for DT-native networks to realize 6G self-evolution. Specifically, a key concept of “future shots” is introduced into the architecture for predicting future states and validating evolution performance. Moreover, we give potential implementation directions and strategy generation methods, including a network evolution strategy, full-scale network prediction method with CTHGAM model and LTHCGAM model for predicting network states and strategy performance, and hierarchical network interaction strategies for efficient virtual–physical interactions. These potential implementation directions have only been theoretically proven feasible, lacking specific practical parameters and simulation validation. In the future, we will further validate the performance of the proposed methods through system-level simulations, which will enhance the feasibility of 6G evolution approaches based on DTs. In the final section of this paper, we give four potential applications for DT-native networks.

## Figures and Tables

**Table 1:** List of abbreviations.

Abbreviation	Full Form
AAU	Active Antenna Unit
AGI	Artificial General Intelligence
ALSSSG	Autonomous Large–Small-Scale Strategy Generation
AMF	Access and Mobility Management Function
CTHGAM	Cross-Time Hierarchical Graph Attention Model
CU	Centralized Unit
DLSTP	Differentiating Long–Short-Term Prediction
DT	Digital Twin
DU	Distributed Unit
FCN	Fully Connected Network
GNN	Graph Neural Network
HGNN	Heterogeneous Graph Neural Network
KPI	Key Performance Indicator
LDT	Local Digital Twin
LLM	Large Language Model
LSTSC	Large–Small Time-Scale Controls
LTHCGAM	Long-Term Hierarchical Convolutional Graph Attention Model
NDT	Network Digital Twin
OAM	Operations, Administration, and Maintenance
RAN	Radio Access Network
RIS	Reconfigurable Intelligent Surface
RL	Reinforcement Learning
RNN	Recurrent Neural Network
SON	Self-Organizing Network
THz	Terahertz
UAV	Unmanned Aerial Vehicle
UPF	User Plane Function

## Data Availability

Data are contained within the article.

## Appendix A. Time Alignment Based on Positional Encoding

(A1) $$\begin{array}{cc} \; & \mathrm{TA}\left(\left(S_{n,i-1},P_{n,i-1},\zeta_{n,i-1}\right),\tau_{n,i-1}^{\mathrm{now}}\right)=\mathrm{FCN}\left(S_{n,i-1},P_{n,i-1},\zeta_{n,i-1}\right)+\left\{\sin(\tau_{n,i-1}^{\mathrm{now}}/10,000^{2j/d})\right\}{|}_{j=1,\ldots ,d}\\ \; & \mathrm{TA}\left(\left(S_{n,i-1},P_{n,i-1},\zeta_{n,i-1}\right),\tau_{n,i-1}^{\mathrm{now}}\right)=\mathrm{FCN}\left(S_{n,i-1},P_{n,i-1},\zeta_{n,i-1}\right)+\left\{\cos(\tau_{n,i-1}^{\mathrm{now}}/10,000^{2j/d})\right\}{|}_{j=1,\ldots ,d} \end{array}$$

where *d* is the dimension of the property vector, *i* denotes different elements in the input vector, and a constant 10,000 ensures that the sine/cosine function wavelengths transition smoothly across different dimensions *j*, covering a range from short to long periods, thereby capturing the relationships between the time scales of different network elements.
